# Riskanter Krankenhausplan – eine Umfrage unter ChefärztInnen in NRW: dramatische Auswirkungen in der Viszeralchirurgie

**DOI:** 10.1007/s00104-025-02253-8

**Published:** 2025-02-12

**Authors:** Chris Braumann, Kirsten Meurer, Marco Niedergethmann, Karl-Heinz Bauer, Alexis Ulrich, Florian Gebauer, Emile Rijcken, Uta Bultmann, Felicitas Giuliana Held, Björn Schmitz, Jasmina Hahn, André Schilling, Andreas Pascher, Konstantinos Zarras, Waldemar Uhl

**Affiliations:** 1 Klinik für Allgemein-Viszeral- und Tumorchirurgie, Evangelisches Krankenhaus Herne, Wiescherstr. 24, 44623 Herne, Deutschland; 2 Klinik für Allgemein- und Viszeralchirurgie, St. Josef-Hospital Bochum, Ruhr-Universität Bochum, Bochum, Deutschland; 3 Klinik für Allgemein- und Viszeralchirurgie, Alfried Krupp Krankenhaus Rüttenscheid, Essen, Deutschland; 4 Knappschaft-Kliniken GmbH, Geschäftsbereich Prävention und Sportmedizin, Deutschland; 5 Klinik für Allgemein- und Viszeralchirurgie, Lukas Krankenhaus Neuss, Neuss, Deutschland; 6 Klinik für Allgemein- und Viszeralchirurgie am Helios Universitätsklinikum Wuppertal, Wuppertal, Deutschland; 7 Knappschaftskrankenhaus, Klinik am Park in Lünen, Klinikum Westfalen, Lünen, Deutschland; 8 Klinik für Allgemein‑, Viszeral- und Thoraxchirurgie, Klinikum Leverkusen, Leverkusen, Deutschland; 9 Klinik für Allgemeinchirurgie, Viszeral und Endokrine Chirurgie, Proktologie, Knappschaftskrankenhaus Dortmund, Dortmund, Deutschland; 10 Klinik für Allgemein‑, Viszeral und Transplantationschirurgie, Universitätsklinikum Münster, Münster, Deutschland; 11 Klinik für Viszeral‑, Minimalinvasive- und Onkologische Chirurgie, Marien Hospital Düsseldorf, Düsseldorf, Deutschland

**Keywords:** Krankenhausreform NRW, Leistungsgruppen, Viszeralchirurgie, Zertifizierungen, Strukturqualität, NRW hospital reform, Service groups, Visceral surgery, Certification, Structural quality

## Abstract

**Hintergrund:**

Der Krankenhausplan NRW soll im größten deutschen Bundesland die medizinische Qualität und die Effizienz der Gesundheitsversorgung steigern respektive optimieren und könnte als „Blaupause“ für Deutschland dienen. Die Feststellungsbescheide des Landesministeriums für Arbeit, Gesundheit und Soziales (MAGS) für die Leistungsgruppen (LG) wurden den Kliniken zugestellt und die definitive Umsetzung für April 2025 vorgesehen.

**Methodik:**

Insgesamt 254 viszeralchirurgischen ChefärztInnen (CÄ) wurde ein konsentierter Fragebogen zugesandt: Alter, Bettenanzahl, Versorgungsstufen, Leistungsgruppen, Zentralisierung, Spezialisierung, Zertifizierung, Änderung der öffentlichen Wahrnehmung, Weiterbildung und Gefahren für die Weiterbildungsbefugnis wurden ebenso wie Attraktivität des Faches und Nachwuchsthemen abgefragt.

**Ergebnisse:**

Von 254 Bögen wurden 108 rückgesendet (42,5 %). 50 % der CÄ sind von der Notwendigkeit der Reform überzeugt. Jedoch ist 32 % der Kliniken die Zuteilung einer LG trotz bestehender Zertifizierung verwehrt worden. Bei 45 Kliniken (42 %) erfolgte gar keine LG-Zuteilung. Ein Krankenhaus erhielt ohne Antragstellung eine LG. 80 Klinikleitungen können keine sinnvolle politische Strategie im Krankenhausplan erkennen (74 %). 35 % der CÄ sehen keine Verbesserung der Behandlungsqualität durch die Zentralisierung. 59 Leitungen befürchten, dass das öffentliche Ansehen ihrer Klinik verschlechtert wird (55 %). 86 CÄ sehen die derzeitige Weiterbildungsordnung nicht mehr garantiert (80 %). 72 % sehen einen Verlust der Attraktivität des Faches Viszeralchirurgie und 48 % der CÄ machen sich Sorgen um ihre persönliche Zukunft.

**Diskussion:**

Die Reform wird von den Befragten unterstützt – die Methode stößt jedoch auf große Kritik. Zertifizierungen der DKG und DGAV finden keine verlässliche Berücksichtigung und zweifeln demnach Lebens- und Klinikleistungen an. Zudem wird eine differenzierte Auswirkungsanalyse und eine Begleitforschung vermisst (z. B. Kapazitätsengpässe, Personalmangel, Wartezeiten, Krankenhausschließungen). Die Attraktivität des Faches Chirurgie ist wie die Zusatzweiterbildung Spezielle Viszeralchirurgie gefährdet.

**Graphic abstract:**

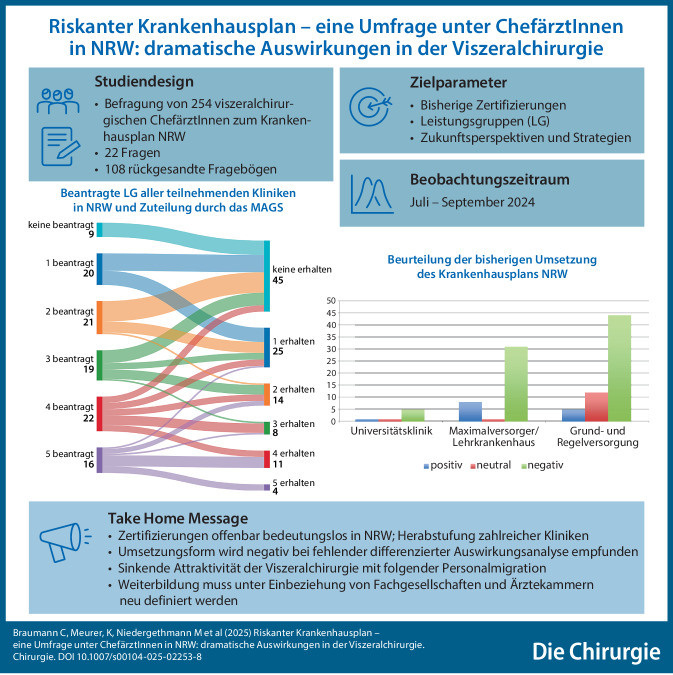

## Abstract (en)

**Background:**

The hospital plan for North-Rhine/Westphalia (NRW) aims to enhance and optimize the quality and efficiency of healthcare in Germany’s largest federal state and could potentially serve as a blueprint for the entire country. The State Ministry of Labor, Health and Social Affairs (MAGS) has issued determination notices for the service groups (LG), which were sent to hospitals with final implementation scheduled for April 2025.

**Method:**

A consensus-based questionnaire was distributed to 254 chief physicians of visceral surgery departments (CÄ). The survey queried information on age, number of beds, care levels, service groups, centralization, specialization, certification, changes in public perception, continuing education and threats to training authorization as well as the attractiveness of the discipline and concerns regarding junior staff.

**Results:**

Out of 254 questionnaires 108 were returned (42.5%). Approximately 50% of the respondents believe the reform is necessary; however, 32% of clinics were denied allocation of an LG despite having certification. In 45 clinics (42%) no LG was allocated at all. One hospital received an LG without applying for it. Additionally, 80 hospital administrators (74%) reported not seeing any meaningful political strategy behind the hospital plan, 35% of CÄs did not anticipate improvements in treatment quality from centralization, 59 head physicians (55%) expressed concerns that their hospital’s public image would deteriorate and 86 CÄs (80%) believed the current training regulations can no longer be guaranteed. Furthermore, 72% of respondents felt that visceral surgery is losing its appeal as a discipline and 48% were worried about their personal future career prospects.

**Discussion:**

While the reform is generally supported by the respondents, the implementation method has received significant criticism. The certifications from the German Cancer Society (DKG) and the German Society for General and Visceral Surgery (DGAV) are not consistently recognized, raising doubts about the reliability of hospital services. Additionally, a detailed impact analysis and accompanying research are lacking (e.g., capacity bottlenecks, staff shortages, waiting times, hospital closures). The attractiveness of surgery as a discipline and advanced training in special visceral surgery are at risk.

## Hintergrund

Die aktuelle Krankenhausplanung in Nordrhein-Westfalen (NRW) hat als politisches Ziel, eine bedarfsgerechte, effiziente und moderne Krankenversorgung sicherzustellen. Die vorhandenen Ressourcen sollen optimal genutzt und die Qualität der medizinischen Versorgung erhöht werden [1].

In NRW gibt es derzeit 328 Krankenhäuser mit ca. 112.610 Betten, wobei 20,4 % der Krankenhäuser > 500 Betten aufweisen. Das Landesministerium für Arbeit, Gesundheit und Soziales (MAGS) des Landes NRW führt seit 6 Jahren eine Reform der Krankenhauslandschaft durch. Der neue Krankenhausplan wurde am 27.04.2022 veröffentlicht. Es wurde ein regionales Planungsverfahren initiiert, welches das bevölkerungsreichste Bundesland modernisieren soll [2–4]. Es ist ein formalisierter Prozess, der Krankenhausträger, Krankenkassen, Ärztekammer und kommunale Spitzenverbände aufforderte, den Bedarf zu analysieren und Konzepte dem MAGS vorzulegen. Auch PatientInnenverbände wurden einbezogen – die chirurgischen Fachgesellschaften, wie beispielsweise die Deutsche Gesellschaft für Chirurgie, die Deutsche Gesellschaft für Allgemein- und Viszeralchirurgie oder die regionalen Fachgesellschaften – in NRW die Niederrheinisch-Westfälische Gesellschaft für Chirurgie –, wurden in dieses Verfahren nicht einbezogen. Im Fokus stehen dabei komplexe Leistungen, die bezüglich ihrer Spezifikation innerhalb der Viszeralchirurgie als fünf Leistungsgruppen (LG) ausschließlich über bestimmten OPS-Codes definiert wurden: bariatrische Chirurgie (LG 16.1), Lebereingriffe (LG 16.2), Ösophaguseingriffe (LG 16.3), Pankreaseingriffe (16.4) und tiefe Rektumeingriffe (16.5; [5, 6]; Tab. 1).

**Tab. 1:** Eine Neuerung des Krankenhausplans NRW ist eine Planungssystematik, in der statt 22 Fachabteilungen nunmehr 32 Leistungsbereiche mit 64 Leistungssubgruppen ausgewiesen werden [12]. Für die Viszeralchirurgie gilt:

Viszeralchirurgie	16.1	Bariatrische Chirurgie	OPS + ICD	E66 UND	Adipositas (nur Hauptdiagnose)
				5‑434.3	Biliopankreatische Diversion nach Scopinaro
				5‑434.4	Biliopankreatische Diversion mit Duodenal-Switch
				5‑434.5	Herstellung eines Schlauchmagens („sleeve resection“)
				5‑434.6	Duodenal-Switch mit Bildung eines gemeinsamen Dünndarmschenkels („common channel“) nach Herstellung eines Schlauchmagens
				5‑445.4	Gastroenterostomie ohne Magenresektion (Bypassverfahren): mit Staplernaht oder Transsektion (bei Adipositas), mit Gastrojejunostomie durch Roux-Y-Anastomose
				5‑445.5	Gastroenterostomie ohne Magenresektion (Bypassverfahren): mit Staplernaht oder Transsektion (bei Adipositas), mit Gastrojejunostomie analog Billroth II
	16.2	Lebereingriffe	OPS	5‑502	Anatomische (typische) Leberresektion
	16.3	Ösophaguseingriffe	OPS	5‑423	Partielle Ösophagusresektion ohne Wiederherstellung der Kontinuität
				5‑424	Partielle Ösophagusresektion mit Wiederherstellung der Kontinuität
				5‑425	(Totale) Ösophagusektomie ohne Wiederherstellung der Kontinuität
				5‑426	(Totale) Ösophagektomie mit Wiederherstellung der Kontinuität
				5‑427.0	Rekonstruktion der Ösophaguspassage (als selbständiger Eingriff) – im Retrosternalraum (vorderes Mediastinum)
				5‑427.1	Rekonstruktion der Ösophaguspassage (als selbständiger Eingriff) – im Ösophagusbett (hinteres Mediastinum)
				5‑429.p	Andere Operationen am Ösophagus – Implantation oder Wechsel eines magnetischen Antirefluxsystems
				5‑429.q	Andere Operationen am Ösophagus – Revision oder Entfernung eines magnetischen Antirefluxsystems
				5‑438.0	(Totale) Gastrektomie mit Ösophagusresektion – Mit (sub)totaler Ösophagusresektion, mit Dünndarminterposition
				5‑438.1	(Totale) Gastrektomie mit Ösophagusresektion – Mit (sub)totaler Ösophagusresektion, mit Dickdarminterposition
				5‑438.x	(Totale) Gastrektomie mit Ösophagusresektion – Sonstige
	16.4	Pankreaseingriffe	OPS	5‑524.0	Partielle Resektion des Pankreas – Linksseitige Resektion (ohne Anastomose)
				5‑524.1	Partielle Resektion des Pankreas – Partielle Duodenopankreatektomie mit Teilresektion des Magens (Operation nach Whipple)
				5‑524.2	Partielle Resektion des Pankreas – Pankreaskopfresektion, pyloruserhaltend
				5‑524.3	Partielle Resektion des Pankreas – Pankreaskopfresektion, duodenumerhaltend
				5‑524.4	Partielle Resektion des Pankreas – Pankreassegmentresektion
				5‑524.x	Partielle Resektion des Pankreas – Sonstige
				5‑525.0	(Totale) Pankreatektomie – Mit Teilresektion des Magens
				5‑525.1	(Totale) Pankreatektomie – Pyloruserhaltend
				5‑525.2	(Totale) Pankreatektomie – Duodenumerhaltend
				5‑525.3	(Totale) Pankreatektomie – Entfernung eines Pankreastransplantates
				5‑525.4	(Totale) Pankreatektomie – Pankreatektomie postmortal (zur Transplantation)
				5‑525.x	(Totale) Pankreatektomie – Sonstige
	16.5	Tiefe Rektumeingriffe	OPS	5‑484.2	Tubuläre Resektion unter Belassen des Paraproktiums
				5‑484.5	Tiefe anteriore Resektion
				5‑484.6	Tiefe anteriore Resektion mit peranaler Anastomose
				5‑485	Rektumresektion ohne Sphinktererhaltung

Im Juni 2024 wurden die Bescheide zugesandt. Nach Ablauf der Widerspruchsfrist wurden die Schreiben des zweiten Anhörungsverfahrens verschickt. Hierbei wurden die ursprünglich zugeteilten LG nochmals deutlich reduziert. Am 16.12.2024 wurden die Feststellungsbescheide den Krankenhausträgern transparent für NRW zugesandt, welche dann ab dem 01.04.2025 in Kraft treten werden [7]. Diese ordnungspolitischen Bescheide bedeuten neben juristischer Untersagung von Leistungen in den betroffenen Krankenhäusern einen deutlichen finanziellen Verlust bei Wegfall der LG. Gerade angesichts der Tatsache, dass in einer aktuellen Erhebung 79 % der Häuser 2024 finanziell rote Zahlen schreiben werden [8], treffen diese Bescheide die Krankenhäuser in einer maximal vulnerablen Phase.

Unsere Umfrage soll Transparenz zur Planung/Anhörung des MAGS in NRW im Hinblick auf die Qualität, Angemessenheit und individuelle Prüfung schaffen und eine Abschätzung der Konsequenzen für die chirurgische Versorgung in NRW und folgend in allen anderen Bundesländern ermöglichen, wenn der Krankenhausplan NRW als sogenannte „Blaupause“ für Deutschland genommen werden sollte.

## Material und Methode

Die Erhebung stellt die Ergebnisse einer repräsentativen Umfrage bei viszeralchirurgischen CÄ in NRW nach Eingang der ersten vorläufigen Bescheide dar. Die Datenbank der Niederrheinisch-Westfälischen Gesellschaft für Chirurgie wurde zu diesem Zweck aktualisiert und genutzt. Die Umfrage umfasst 22 Fragen, welche vom Vorstand in einem Konsensfindungsverfahren formuliert wurden. Jeder Chefarzt/Chefärztin wurde dreimalig kontaktiert, um den Rekrutierungsanteil zu erhöhen.

Die IP-Adressen wurden vom Programm SurveyMonkey Umfrage-Tool mit integriertem Analysemodul registriert, sodass Mehrfachnennungen nicht möglich waren. Ein Überspringen von Teilbereichen war nicht zulässig. Bei Abschluss wurden alle Antworten an einen Rechner weitergeleitet, auf welchem die Daten anonymisiert ausgewertet wurden (SurveyMonkey im Stellenwert-Zahlensystem zur Basis 10). Die graphischen Abbildungen wurden durch Säulen‑, Balken- und Swimlane-Charts durch SankeyMATIC ausgewertet und visualisiert.

Die konsentierte Interpretation, Wichtung und Bewertung erfolgte durch die Mitglieder des Vorstandes und des erweiterten Vorstandes (Beirates).

## Ergebnisse

Insgesamt 254 Krankenhäuser mit einer Allgemein- und Viszeralchirurgie wurden ab Juli 2024 angeschrieben. 108 vollständige Antwortbögen (42,5 %) wurden bis zum 15.09.2024 elektronisch registriert. Es liegen die Bögen von 7 Universitätskliniken, 40 Kliniken der Maximalversorgung und 61 Grund- und Regelversorgungskrankenhäusern vor. Ausgewertet wurden Rückmeldungen von 101 männlichen und 7 weiblichen Teilnehmenden (Frage **F1, 2** und **4**).

### F3 *Wie lange sind Sie Chefarzt/Chefärztin? Erfahrungsspektrum in der Funktion als Chefarzt/Chefärztin*?

Weniger als 5 Jahre sind 24 % als CÄ tätig. 32 % sind zwischen 6 und 10 Jahre in dieser Position angestellt. 16 % bekleiden das Amt zwischen 11 und 15 Jahren. 28 % der CÄ sind über 16 Jahre tätig.

### F5 *Wie viele Betten hat Ihr Krankenhaus?*

Insgesamt 9 Krankenhäuser besitzen > 800 Betten. Eine Krankenhausgröße mit 250 bis 500 Betten findet sich bei der Hälfte (49 %). 23 Krankenhäuser (21 %) verfügen über < 250 Betten und ebenso viele zwischen 500 und 800.

### F6 *Wie viele Betten hat Ihre Abteilung oder Klinik?*

**Abb. 1:**
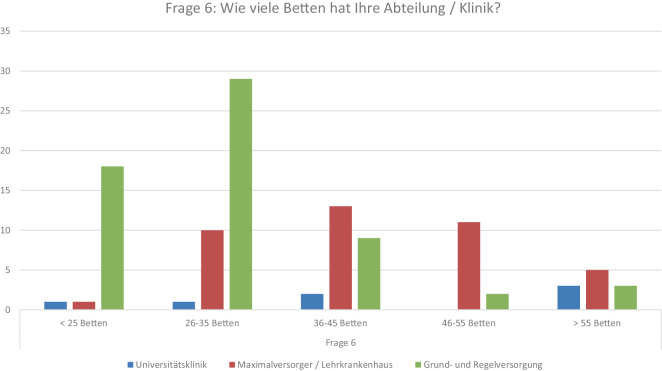
Frage 6: Wie viele Betten hat Ihre Abteilung/Klinik?

Zwischen 26 und 35 Betten gaben 40 Kliniken an (37 %). 24 Kliniken verfügen über bis zu 45 Betten (22 %). Nur 11 Abteilungen haben > 55 Betten, wohingegen 20 Kliniken < 25 Betten angaben (Abb. 1).

### F7 *Wie viele Leistungsgruppen haben Sie beantragt?*

Es konnten bis zu 5 LG in der Viszeralchirurgie beantragt werden. 9 Krankenhäuser hatten keine LG beantragt. 17 Krankenhäuser wünschten 5 LG, die meisten Institutionen (*n* = 22) forderten 4 LG (20 %).

### F8 *Wie viele Leistungsgruppen haben Sie zugesprochen bekommen?*

Insgesamt 45 Kliniken haben keine LG zugesprochen bekommen (42 %). 4 Krankenhäuser dürfen 5 LG anbieten (4 %). 26 Kliniken werden 1 LG behalten (24 %), wohingegen 14 Krankenhäuser 2 LG versorgen dürfen (13 %). 11 Krankenhäuser haben 4 LG. 8 Kliniken werden 3 LG anbieten (7 %).

### F9 *Haben Sie eine Leistungsgruppe zugewiesen bekommen, die nicht beantragt wurde?*

Eine Klinik erhält 1 LG ohne Ersuchen.

### F10 *Wurde eine beantragte Leistungsgruppe abgelehnt, auch wenn dafür eine entsprechende Zertifizierung, der DGAV oder der DKG als Zentrum vorlag?*

Bei 32 Kliniken wurden Anträge abgelehnt (30 %). 76 zertifizierte Kliniken erhalten Zuteilungen (70 %).

### F11 *Wie beurteilen Sie grundsätzlich den Krankenhausplan NRW mit Zentralisierung bestimmter Leistungen?*

Es sind 50 CÄ von der Sinnhaftigkeit überzeugt (46 %), wohingegen sich 42 Kliniken negativ äußern (39 %). 16 CÄ sind unschlüssig (15 %).

### F12 *Wie beurteilen Sie die bisherige Umsetzung des Krankenhausplans NRW?*

**Abb. 2:**
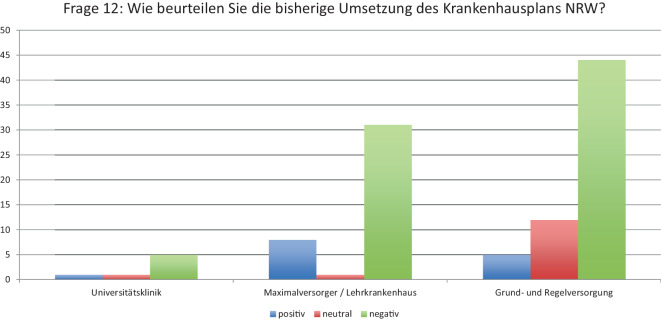
Frage 12: Wie beurteilen Sie die bisherige Umsetzung des Krankenhausplans NRW?

Insgesamt 80 CÄ können keine sinnvolle politische Strategie erkennen (74 %). 14 Klinikleitungen sehen positive Ansätze, wohingegen sich ebenso viele neutral verhalten (Abb. 2).

### F13 *Glauben Sie, dass sich die Versorgungsqualität durch eine Zentralisierung und zentrale Steuerung verbessern lässt?*

Insgesamt 38 CÄ sehen keine Verbesserung durch politische Steuerung (35 %). Jeweils 35 Kliniken beurteilen dieses konträr (32 %). Ebenso viele können keine bessere Begründung finden.

### F14 *Glauben Sie, dass die PatientInnen in NRW von der Reform profitieren?*

Keinen Nutzen für Patienten können 54 CÄ (50 %) erkennen. 29 geben an, Vorteile ableiten zu können (27 %). 29 CÄ haben kein vollständiges Vertrauen in die Reform und sind zurückhaltend (27 %).

### F15 *Glauben Sie, dass sich die öffentliche Wahrnehmung Ihres Krankenhauses ändern wird?*

Insgesamt 59 Klinikleitungen befürchten ein verschlechtertes Ansehen (55 %), wohingegen 19 mit einer Stabilisierung rechnen (18 %). 30 CÄ erwarten keine Änderung (27 %).

### F16 *Glauben Sie, dass sich die öffentliche Wahrnehmung Ihrer Abteilung oder Klinik ändern wird?*

Eine Verschlechterung des Ansehens vermuten 62 Leitungskräfte (58 %), 20 CÄ sehen positives Potenzial (18 %). 26 erwarten keine Änderung (24 %).

### F17 *Wird sich die chirurgische Weiterbildung in der jetzt gültigen Fassung in Zukunft in NRW noch realistisch aufrechterhalten lassen?*

**Abb. 3:**
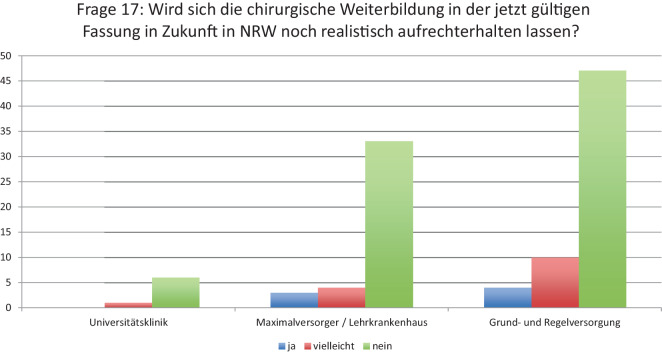
Frage 17: Wird sich die chirurgische Weiterbildung in der jetzt gültigen Fassung in Zukunft in NRW noch realistisch aufrechterhalten lassen?

Insgesamt 86 CÄ sehen die derzeitige Weiterbildungsform nicht als gesichert an (80 %). 15 Klinikleitungen sehen Chancen durch die Reform (14 %), wogegen 7 keine Effekte erwarten (6 %, Abb. 3).

### F18 *Sehen Sie Ihre Weiterbildungsbefugnis in Gefahr?*

Den Verlust ihrer bisherigen Weiterbildungsbefugnis befürchten 68 CÄ (63 %). 22 Klinikleitungen sehen keine Zukunftsänderungen (20 %), wohingegen 18 CÄ die Effekte nicht abschätzen können (17 %).

### F19 *Wird die Attraktivität des Faches für Chirurgie und damit die Nachwuchsgewinnung unter der Reform leiden?*

Insgesamt 72 % der CÄ befürchten einen Verlust der Attraktivität der Viszeralchirurgie. 11 Klinikleitungen sind weiterhin von der Attraktivität überzeugt (10 %). 19 CÄ können es nicht bewerten (18 %).

### F20 *Fühlen Sie sich in Ihrer Berufsausübung als ÄrztIn durch die Einführung der zentralen Vergabe der Leistungsgruppe beeinträchtigt?*

Insgesamt 89 Befragte gaben an, sich durch die zentrale Vergabe der Leistungsgruppen beeinträchtigt zu fühlen (82 %), wobei sich 19 Klinikleitungen nicht kompromittiert erleben (18 %).

### F21 *Machen Sie sich Sorgen um Ihre persönliche Zukunft, falls die Reform in dieser Form umgesetzt wird?*

Persönliche Zukunftssorgen beschreiben 52 CÄ (48 %). 56 Klinikleitungen sind nicht beunruhigt (52 %).

### F22 *Denken Sie über eine frühzeitigere Berufsaufgabe nach, falls die Reform in dieser Form umgesetzt wird?*

Über eine vorzeitige Berufsaufgabe denken 42 Klinikleitungen nach (39 %). 66 CÄ sind unbeeindruckt (61 %).

**Abb. 4:**
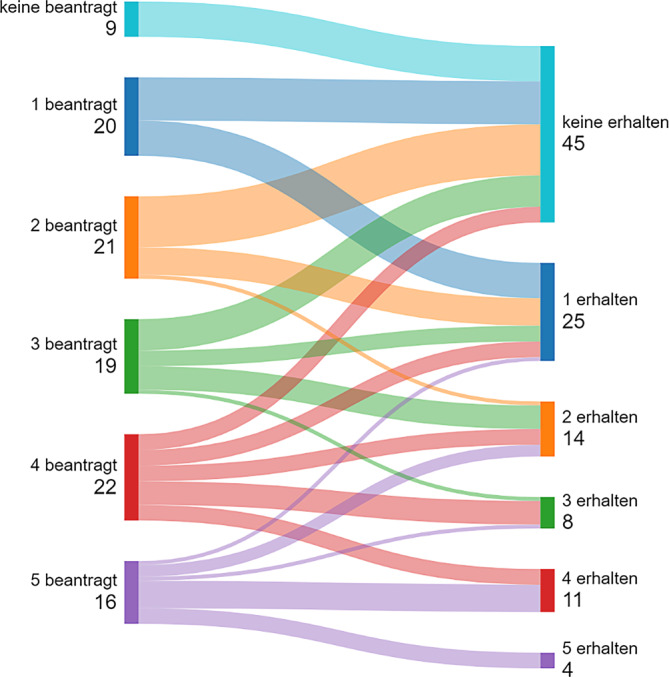
Beantragte LG aller antwortenden Kliniken in NRW und Zuteilung/Erhalt (Swimlane-Chart). *Links* Selbsteinschätzung der Krankenhäuser und Antragsumfang/LG; *rechts* Zuteilungsausmaß durch das MAGS. Ungeachtet der bisherigen Qualität und Leistung bzw. des geographischen Standorts wird eine massive Umstrukturierung der Krankenhauslandschaft folgen

**Abb. 5:**
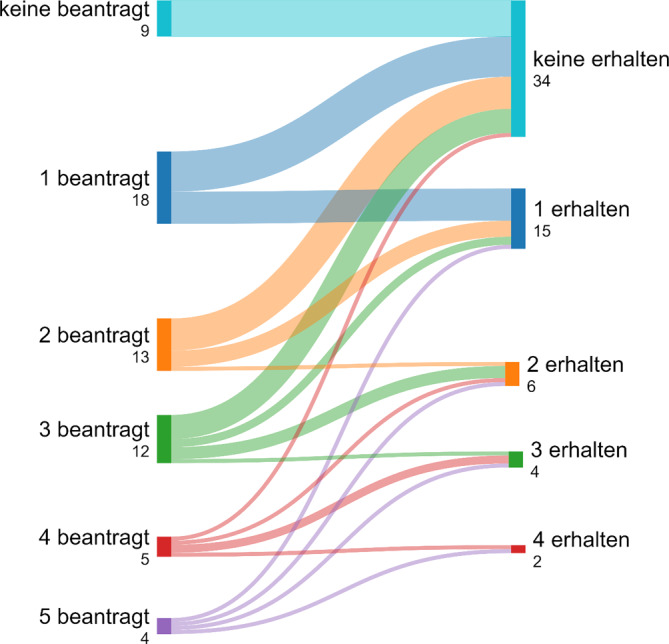
LG-Zuteilung der Krankenhäuser der Grund- und Regelversorgung (Swimlane-Chart). Visualisierung der Subpopulationen bezüglich der beantragten LG (*links*) sowie der resultierenden Zuteilungen (*rechts*). Ist eine Degradierung zu erkennen, so sind beantragte LG vom MAGS abgesprochen worden (*ansteigende Kurve*). Ist eine *horizontale Linie* ersichtlich, so entspricht die Anzahl der zugeteilten LG der Anzahl der beantragten LG. Die *Swimlane-Stärke* beinhaltet die Menge/Anzahl. Überwiegend liegt eine Herabstufung vor. Die maximal zugeteilte Menge beträgt 4 LG. Fünf Krankenhäuser haben 4 Spezialisierungen beantragt: eine Klinik hat diese erhalten, wohingegen eine Klinik ohne Berücksichtigung blieb. Häufig wurde nur 1 LG vergeben. Meist wurden 2 und 3 LG beantragt, die vorwiegend verwehrt wurden. Die Mehrheit der Grund- und Regelversorger erhielt keine Zuteilung

Die Swimlane-Charts in den Abb. 4, 5, 6 und 7 beschreiben eine longitudinale Betrachtung und stellen den beantragten LG die Zuteilungen des MAGS gegenüber. Unterhalb der Abbildungslegenden erfolgt die Charakteristik.

**Abb. 6:**
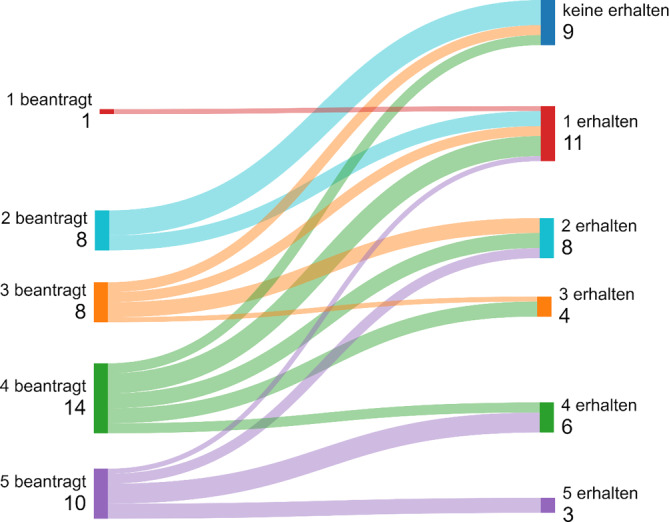
LG-Zuteilung der antwortenden Maximalversorger (Swimlane Chart). Beantragung von LG-Anzahl (*links*), Anzahl Klinikanträge *untere Zeile links*; Zuteilung durch das MAGS *rechts*. Die maximalversorgenden Kliniken beantragten mehr LG als die Häuser der Grund- und Regelversorgung. Je höher der Anstieg der Swimlane-Kurve, desto größer die Anzahl an verwehrten Zuteilungen. So ist bei einer Klinik die Diskrepanz von 5 beantragten LG zu lediglich 1 Zuteilung dokumentiert. Die Mehrzahl der Maximalversorger wurde auf 4 bzw. 2 LG gestuft. 14 Kliniken wollten 4 LG anbieten, 3 dieser Kliniken wurden auf 3 sowie auf 2 Spezialisierungen gestuft, 2 Kliniken erhielten keine Zuteilung. Acht Kliniken wünschten die Berücksichtigung von 3 LG: 2 Kliniken können 1 LG und 2 weitere Kliniken keine LG anbieten

**Abb. 7:**
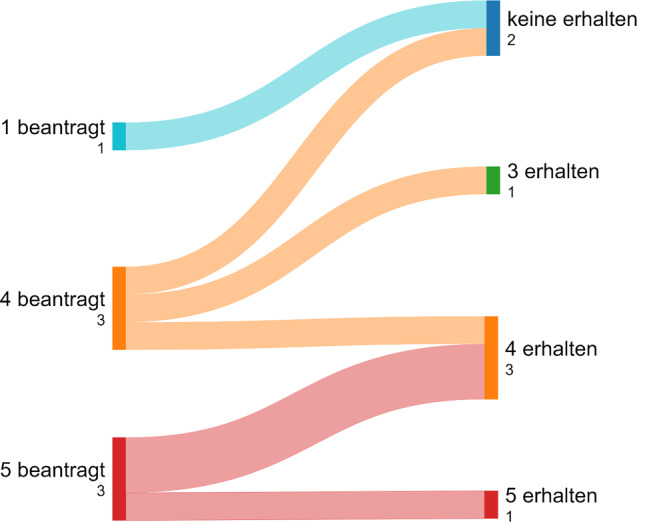
Universitäre Beantragungen von LG (Swimlane-Chart). Zwei universitäre Kliniken erhielten die umfänglich beantragten 5 und 4 LG. Zwei universitäre Häuser beantragten 1 oder 4 LG und wurden nicht gewürdigt. In den übrigen Fällen wird 1 LG berichtet

## Diskussion

*Alea iacta est!* Der Feststellungsbescheid in NRW verfolgt das Ziel einer „Qualitätsverbesserung durch Leistungskonzentration, Kooperation sowie Ressourcennutzung“.

Unsere aktuelle Umfrage offeriert ein authentisches Stimmungsbild der CÄ in der Allgemein- und Viszeralchirurgie in NRW. Die Teilnahme ist aufgrund des knappen Befragungszeitraums mit 40 % überaus erfolgreich zu werten. Obwohl sich 82 % in ihrer freien Berufsausübung teils beschnitten fühlen, schließt sich ein beachtlicher Teil eines Reformgedankens kompromissbereit an. 50 % sind von der Notwendigkeit einer Umorganisation überzeugt, jedoch wird der Realisierungsversuch von 80 % als inadäquat bezeichnet. Die Diskrepanz zwischen Konzeption und Umsetzungswunsch signalisiert eine ungewisse Zukunft. Daher denken 39 % über einen Berufsausstieg nach.

Wo findet sich hier der Grundgedanke der Anhebung von Struktur‑, Prozess- und Versorgungsqualität? Die Grundlage der Berechnung waren „Zahlen“ vor der COVID-Pandemie – also 2019. Einige Kliniken beantragten inadäquat hohe Fallzahlen. Überraschend wurden diese teils vom MAGS übernommen, ohne dass die geforderte Struktur- und Prozessqualität (Ergebnisqualität) individuell geprüft wurde. Unbestritten und bewiesen bleibt, dass hohe Fallzahlen in der Bewältigung von Komplikationen Leben retten. So ist das Komplikationsmanagement der entscheidende Mechanismus für den Zusammenhang zwischen Fallzahl und Behandlungsergebnis. Daher ist es wichtig, dass eine 24/7-Radiologie/Endoskopie zur Verfügung steht, um ein „failure to rescue“ zu reduzieren [9–11]. Überraschend haben Kliniken LG zugeteilt bekommen, obwohl sie die Zertifikate früher nicht mehr erfüllt haben. Dieses konterkariert das zentrale Merkmal „Qualitätsverbesserung“ und würde in wissenschaftlichen Studien schlichtweg untragbar sein und einer Vernehmung nicht standhalten.

Einige Institutionen werden gefördert, andere trotz zuvor erfüllter Qualitätskriterien herabgestuft. Der zugrunde liegende Qualitätsmaßstab ist für CÄ nicht ersichtlich, da dieser nicht nur in Zahlen bemessen werden kann. Ebenso fehlt die Attestierung bisheriger „schlechter Qualität“, insbesondere im Kontext des international anerkannten deutschen Gesundheitssystems durch die meisten Länder. Desgleichen fehlt eine differenzierte Auswirkungs- oder Nebenwirkungsanalyse zu unklaren Sekundär- und Tertiäreffekten. Bei der enormen Bedeutung war es ein unverständlicher Fehler (Wille), keinen ärztlichen Entscheidungsträger zu laden. Die Diskrepanz zwischen dem Zielbild der Reform und der historisch entwickelten Krankenhausstruktur hätte es gefordert.

Die ursprünglich zugeteilten LG wurden vom MAGS bei den endgültigen Feststellungsbescheiden nochmals deutlich reduziert: die LG 16.1 um 60 % auf 29 Kliniken, die LG 16.2 um 76 % auf 26, LG 16.3 um 63 % auf 26 [8], LG 16.4 um 61 % auf 43 und LG 16.5 um 55 % auf 80. Die geforderten Personal- und auch Strukturveränderungen werden mit einem erheblichen Investitionsbedarf und mit der Insolvenz zahlreicher Krankenhäuser einhergehen [8]. Für die Konzentration von „komplexen“ Leistungen müssen unverhältnismäßig zusätzliche Kapazitäten geschaffen werden, da vereinzelte Standorte sogar eine Zuteilungssteigerung erhielten (Ösophagus- und Lebereingriffe jeweils 11 und Rektumeingriffe sogar 25; Universitätsklinik Köln 280 Ösophagus- und St. Josef-Hospital Bochum 255 Pankreaseingriffe). Die Höhe der erforderlichen Investitionen ist nicht bezifferbar und käme bei Einhaltung aller Fristen um Jahre zu spät. Außerdem ist die Ressource Ärzte und Pflege (OP und Intensiv) aktuell bereits ungenügend besetzt. Es ist bekannt, dass Operationssäle und Intensivstationsbetten reduziert betrieben werden. Inwieweit durch Klinikschließungen und -verbünde ein Personaltransfer stattfinden wird, muss abgewartet werden. Es ist jedoch zu befürchten, dass die geburtenstarken Jahrgänge personalneutral ausscheiden und die Situation unverändert bleibt.

Die personelle Grundvoraussetzung für Krankenhäuser setzt sich nach Abschaffung des Facharztes für Chirurgie aus Allgemeinchirurgen, Viszeralchirurgen sowie Unfallchirurgen zusammen. Es ist kaum vorstellbar, dass ein Viszeralchirurg ossäre Frakturen behandelt oder ein Unfallchirurg operative Eingriffe bei einem Kolonkarzinom oder Magenulkus durchführt. Den europäischen Allgemeinchirurgen gibt es in Deutschland (noch) nicht, eine spätere Musterweiterbildungsordnung könnte diesem jedoch Rechnung tragen. Für diese Umsetzung wird es aber Jahre brauchen.

Die aktuelle politische Entscheidung wird bei einigen Dienstgebern vermehrt zu wirtschaftlicher Insolvenz, Stagnation in der Patientenbehandlung, zu Personalmigration sowie zur Beeinträchtigung beruflicher Perspektiven führen. Damit wird die Weiterbildung (WB) der Ärzte gefährdet und, falls unverändert, der behördliche Grundgedanke für die weiteren Bundesländer kein Vorbild. Mit Beginn einer neuen Ära spielt für derzeitige CÄ der Zukunftsaspekt der Fachdisziplin eine entscheidende Rolle. Dieses Feld darf dem G‑BA jedoch nicht überlassen werden. Die Ärztekammern und die DGAV sind daher zur vertieften Zusammenarbeit aufgerufen, um eine neue Definition des viszeralchirurgischen Facharztes oder Alternativen zu entwerfen.

Eine Möglichkeit wäre die Orientierung an europäischen oder angloamerikanischen WB-Modellen (Individualist/Spezialist: hepatopankreatikobiliäre Chirurgie, oberer Gastrointestinaltrakt, unterer Gastrointestinaltrakt oder kolorektale Chirurgie, bariatrische Chirurgie, Bauchwandhernienchirurgie, endokrine Chirurgie, Transplantation), welche den bisher zusammenfassenden Facharzt für Spezielle Viszeralchirurgie durch Module ersetzen würden und den „Generalisten“ verliert. Nachfolgend würde die generelle viszeralchirurgische Expertise nicht zur Verfügung stehen und wahrscheinlich in bekanntem Umfang auch nicht mehr auszubilden sein. Damit würde die Ära des ganzheitlich orientierten, umfassend und wissenschaftlich geformten Chirurgen beendet werden. Dies steht in deutlichem Gegensatz zu den zukunftsorientierten Formulierungen unseres Beirates „Junge Chirurgie“ der Gesellschaft der Chirurgie NRW, welcher 2025 den Bestand des viszeralchirurgischen Generalisten wünscht. Die WB-Befugnis wird in der jetzigen Form in den meisten Häusern nicht mehr Bestand haben können. Die Strukturreform muss auch eine Änderung der WB induzieren, welche eine zentral definierte WB an verschiedenen Krankenhäusern einer Region impliziert. Ob WB-Verbünde akzeptiert werden, muss abgewartet werden.

Bei der LG 16.2 wurden erschwerenderweise OPS-Codes für „anatomische Resektionen“ herangezogen. Damit wurde eine Reduktion auf 26 Kliniken erreicht. Während für die anderen LG vom G‑BA Mindestmengen vorliegen, gibt es für benannte Lebereingriffe bis dato keine Mindestmenge. Zudem werden hierunter simultane Leberresektionen wie z. B. Metastasenchirurgie nicht abgebildet. Dies hat einen direkten Effekt auf die Umsetzung der für die Darmkrebszentren geforderten Strukturen. Major-Resektionen bleiben hierbei unbestritten. Das Problem für zertifizierte Darmkrebszentren besteht jedoch darin, dass eine Kombination mit einem formell anatomischen Lebereingriff nicht mehr durchgeführt werden darf. Eine Differenzierung in dieser medizinisch-chirurgischen Sachlage wird für einweisende Ärzte schwerlich möglich sein. Somit werden den Darmkrebszentren auch die atypischen Leberresektionen entzogen. Hier wäre adäquater chirurgischer Sachverstand in Beratung durch die Fachgesellschaften essenziell gewesen. Warum die bariatrische Chirurgie eine LG wurde, bleibt wohl ebenso das Geheimnis von MAGS und BMG und imponiert weniger schlüssig als eine Formulierung von z. B. Magen- und Koloneingriffen als LG.

Eine Optimierung der Behandlungsqualität ist offenbar nicht zentraler Bestandteil der politischen Entscheidung, da 30 % der zertifizierten Zentren bei der Zuteilung unberücksichtigt bleiben. So wurden LG bei ca. 50 % der Maximal- und Regelversorger nicht wie beantragt genehmigt. Dies entzieht den Kliniken nicht nur die Würdigung ihrer bisherigen Leistungen, sondern stellt maßgeblich die Kompetenz der chirurgischen Fachgesellschaften und im Falle der Tumorerkrankungen (Ösophagus‑, Pankreas- und Kolorektalkarzinome) die Autorität der Deutschen Krebsgesellschaft in Frage. Formal stellen Reglementierungen ein ordnungspolitisches partielles Berufsausübungsverbot dar, obwohl individuelle Expertise und geforderte Qualitäten bewiesen sind. Zertifizierungen werden hingegen nur noch dort möglich sein, wo die LG zugeteilt sind. Der Entscheidung könnte demnach keine individuelle Prüfung der „Versorgungsqualität“, sondern lediglich ein arbiträres Zuteilungskonzept zugrunde liegen? Die Zentralisierung des Angebots ist ein verständlicher Ansatz. Jedoch wird dabei die flächendeckende Grund‑/Notfallversorgung und der Qualitätswettbewerb gefährdet. Ein konkretes Zielbild ist nicht erläutert worden und dadurch für einige existenzgefährdend. Der Entzug von LG führt aber auch zu sekundären Effekten, d. h. potenzielle Folgeeingriffe (z. B. Portoperationen, Stomabehandlungen, Hernien, Lebermetastasen etc.) werden mutmaßlich nicht in anderen resp. den regionalen Kliniken durchgeführt, sondern werden einen „Klebeeffekt“ bei den Kliniken mit den zugewiesenen LG haben. Tertiäre Effekte werden die Auswirkungen verschärfen, wenn die Spirale der auferlegten Leistungsminderung zu einer weiteren Reduktion der Erlöse für die betroffenen Kliniken führt.

Der 01.04.2025 markiert nun die Umsetzungspflicht des Krankenhausplans NRW. Dies hat erhebliche Konsequenzen bei der Patientenlenkung und der Sicherheit des Arbeitsplatzes. Unklar bleibt, ob CÄ mit Feststellungsbescheid im Falle einer nicht zugeteilten LG strafrechtlich belangt werden könnten. Die juristische Stellungnahme könnte polarisieren und steht noch aus.

Es stellt sich dabei nicht die Frage „ob?“, sondern „wie schnell?“ der Prozess eine Anpassung der Patientenversorgung bewirkt. Als eine besondere Herausforderung ist das Management maligner Erkrankungen anzusehen. Tumorbehandlungen erfordern eine interdisziplinäre Zusammenarbeit, wie sie bereits durch die Zertifizierungsstandards der DKG oder DGAV festgelegt sind. Validierte Studien definieren hierbei das Zeitfenster der chirurgischen Therapie. Eine Konzentration in Kliniken mit LG-Zuteilung wird nachweislich zu Missachtung der geforderten Zeiträume führen, da die Kliniken zum jetzigen Zeitpunkt schlichtweg die erforderliche Personal- und Bettenkapazität nicht vorweisen. Die Überlastung wird nicht nur Tumorkonferenzen, sondern neben Normal- auch IMC- und Intensivstationen (ITS) betreffen. Ist die Kapazitätsgrenze erreicht, so sind Kliniken zur optimierten Patientenversorgung zwingend angehalten, die nächst erreichbare ITS um Übernahme zu bitten. Hierbei wird das Gesundheitssystem an Grenzen stoßen, die bereits jetzt absehbar sind. Ob Klinikverbünde in der Lage sind, die Kapazitätsengpässe durch Verlegung nicht-LG-regulierter Eingriffe an anderen Häusern des Verbundes aufzufangen, bleibt abzuwarten. Die Begleitveränderungen des politischen Willens beunruhigen zudem nachhaltig, da eine umfassende Auswirkungsanalyse fehlt und nicht hauptsächlich auf einer Entfernungsanalyse/Erreichbarkeit basieren darf.

## Ausblick

Eine Reform des Gesundheitswesens in Deutschland ist unbestritten und zwingend notwendig und wird von allen CÄ unterstützt. Beispielhaft besteht Evidenz an der Reduktion des „failure to rescue“ nicht nur durch Fallzahlerhöhung, sondern mit der Bereithaltung einer entsprechenden Struktur- und Prozessqualität für das Komplikationsmanagement. Die Zuteilungen des MAGS sind jedoch arbiträr, nicht individuell und sachverständig geprüft und halten einer Erklärung nicht stand. DKG- und DGAV-Zertifizierungen wurden in einem nicht akzeptablen Ausmaß ignoriert und LG bei ca. 50 % der Maximal- und Schwerpunktversorger nicht mehr genehmigt. Besonders kritisch ist die Garantie definierter Behandlungszeiträume bei Tumorpatienten. Eine differenzierte Auswirkungsanalyse sowie Begleitforschung zu Sekundär- und Tertiäreffekten fehlt und ist aus Sicht der CÄ unabdingbar.

Die vorliegenden Strukturpläne in NRW von Seiten des MAGS gelten als Prototyp für Veränderungen, wie sie in Zukunft in allen deutschen Bundesländern zu erwarten sind („Blaupause“). Daher ist es essenziell, dass die Fachgesellschaften (DGAV mit den regionalen Fachgesellschaften, DKG) mit den Gesundheitsministerien in den Dialog treten, um möglichen Fehlentwicklungen, wie wir sie aufgezeigt haben, entgegenzuwirken, um im Gegenzug eine wirklich flächendeckende Versorgungsqualität für unsere chirurgischen Patienten zu gewährleisten. Es wird dem MAGS sachverständige Beratung durch unsere Fachgesellschaften angeboten, damit das ursprünglich definierte Ziel der Qualitätsanhebung erreicht werden kann. Immer auch vor dem Hintergrund, dass man beim Hausbau nicht das Dach zuerst baut, sondern das wichtige Fundament.

## Data Availability

Die Datensätze können auf begründete Anfrage in anonymisierter Form beim korrespondierenden Autor angefordert werden. Die Daten befinden sich auf einem Datenspeicher im St. Josef-Hospital Bochum, Katholisches Klinikum Bochum der Ruhr-Universität Bochum.
